# Fixation of CO_2_ in *Clostridium cellulovorans* analyzed by ^13^C-isotopomer-based target metabolomics

**DOI:** 10.1186/2191-0855-3-61

**Published:** 2013-10-09

**Authors:** Masahiro Shinohara, Hiroshi Sakuragi, Hironobu Morisaka, Hideo Miyake, Yutaka Tamaru, Eiichiro Fukusaki, Kouichi Kuroda, Mitsuyoshi Ueda

**Affiliations:** 1 Division of Applied Life Sciences, Graduate School of Agriculture, Kyoto University, Sakyo-ku, Kyoto 606-8502, Japan; 2 Kyoto Industrial Science and Technology Innovation Center, Shimogyo-ku, Kyoto 600-8813, Japan; 3 Department of Life Sciences, Graduate School of Bioresources, Mie University, 1577 Kurimamachiya, Tsu, Mie 514-8507, Japan; 4 Department of Biotechnology, Graduate School of Engineering, Osaka University, 2-1 Yamadaoka, Suita, Osaka 565-0871, Japan

**Keywords:** CO_2_ fixation, *Clostridium cellulovorans*, Target metabolomics

## Abstract

*Clostridium cellulovorans* has been one of promising microorganisms to use biomass efficiently; however the basic metabolic pathways have not been completely known. We carried out ^13^C-isotopomer-based target metabolome analysis, or carbohydrate conversion process analysis, for more profound understanding of metabolic pathways of the bacterium. Our findings that pyruvate + oxaloacetate, fumarate, and malate inside and outside cells exhibited ^13^C incorporation suggest that *C. cellulovorans* exactly fixed CO_2_ and partly operated the TCA cycle in a reductive manner. Accompanied with CO_2_ fixation, the microorganism was also found to produce and secrete lactate. Overall, our study demonstrates that a part of *C. cellulovorans* metabolic pathways related to glycolysis and the TCA cycle are involved in CO_2_ fixation.

## Introduction

*C. cellulovorans*, an anaerobic mesophilic bacterium, can degrade and assimilate not only various kinds of carbohydrates (including cellulose, xylan, pectin, cellobiose, glucose, fructose, galactose, and mannose) and but also actual biomass (rice straw and corn waste) (Tamaru et al. 2010b), and whose whole genome was recently sequenced for the first time by our group (Tamaru et al. 2010a). This wide spectrum of degradation depends on extracellular multi-protein complexes called cellulosomes in several cellulosome-producing *Clostridium* species reported; however, most of the researches focus on the cellulosome itself. In order to use *Clostridium* species for practical applications, it is important to elucidate the basic biology of these bacteria, especially their metabolic processes that are highly associated with the conversion of carbohydrates to final products.

*C. cellulovorans* has been suggested to have a CO_2_ fixation pathway, because of its ability to grow under a higher concentration of ‘100%’ CO_2_ compared to other *Clostridium* species (an atmosphere of 20% CO_2_ (*C. cellulovorans*); 5% CO_2_ (*C. acetobutylicum* and *C. kluyveri*); 10% CO_2_ (*C. thermocellum* and *C. difficile*) (Sleat et al. 1984; Amador-Noguez et al. 2010; Waller et al. 2013; Saujet et al. 2011; Thauer et al. 1968). Previously, a few studies have characterized the metabolic pathway of *C. kluyveri* and *C. acetobutylicum* (Jungermann et al. 1970; Amador-Noguez et al. 2010). In the genome analysis of *C. cellulovorans* (Tamaru et al. 2010a), the genes of 2 important CO_2_ fixation enzymes, namely pyruvate:ferredoxin oxidoreductase (PFOR) and phosphoenolpyruvic acid (PEP) carboxylase (PEPC) were annotated. Notably, PFOR of glycolysis and PEPC of the TCA cycle are both in the node of main metabolic pathways in *C. cellulovorans*. Therefore, the study of CO_2_ fixation by metabolome analysis would help to clarify the complete metabolic pathway of *C. cellulovorans*. In particular, ^13^C-labeling studies of metabolic products are useful for understanding the *in vivo* metabolism since 13-carbon isotope can distinguish fluxes through different pathways when these fluxes result in different positional isotopic enrichments in metabolic intermediates (Ratcliffe and Shachar-Hill 2006; McKinlay et al. 2007).

**Figure 1:**
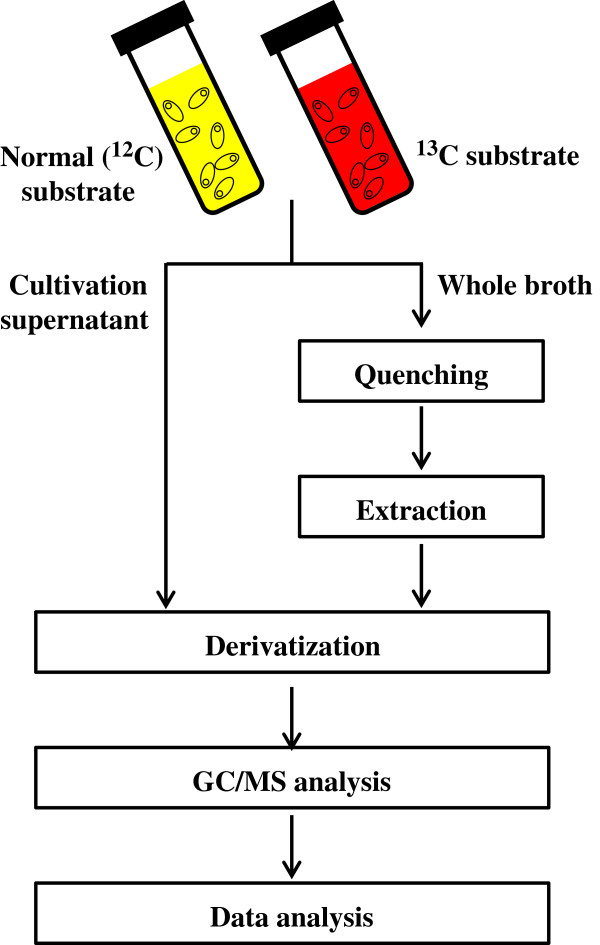
**Workflow for the investigation of CO**_**2 **_**incorporation and the quantification of metabolites of interest.**

As illustrated in Figure 1, we carried out labeling experiments of metabolic intermediates by allowing *C. cellulovorans* to grow in medium with an atmosphere of ‘100%’ CO_2_ containing either NaH^13^CO_3_ or [U-^13^C]-glucose as a labeling reagent, followed by the GC/MS analysis. We demonstrated metabolic fluxes of *C. cellulovorans* and discussed the physiological meaning of CO_2_ fixation in the metabolic pathway of *C. cellulovorans*.

## Materials and methods

### Cultivation conditions and growth rate analysis

*C. cellulovorans* 743B (ATCC 35296) was grown anaerobically at 37°C in an atmosphere of ‘100%’ CO_2_ unless otherwise noted. Liquid cultivation media contained the following reagents: 0.45 g/l KH_2_PO_4_ · H_2_O, 0.45 g/l K_2_HPO_4_, 0.9 g/l NaCl, 0.3675 g/l NH_4_Cl, 0.1575 g/l MgCl_2_ · 6H_2_O, 0.12 g/l CaCl_2_ · 2H_2_O, 5.2 mg/l Na_2_-EDTA, 1.5 mg/l FeCl_2_ · 4H_2_O, 0.942 mg/l CoCl_2_ · 6H_2_O, 0.85 mg/l MnCl_2_ · 4H_2_O, 0.07 mg/l ZnCl_2_ · 6H_2_O, 0.062 mg/l H_3_BO_4_, 0.036 mg/l Na_2_MoO_4_ · 2H_2_O, 0.024 mg/l NiCl_2_ · 6H_2_O, 0.017 mg/l CuCl_2_ · 6H_2_O, 5g/l NaHCO_3_, 4 g/l Bacto™ Yeast Extract (Becton and Dickinson Company), 3 g/l glucose, and 1 g/l L-cysteine. For labeling experiments, NaHCO_3_ and glucose were replaced by NaH^13^CO_3_ and [U-^13^C]-glucose, respectively (both 99% purity; Cambridge Isotope Laboratories, Andover, MA).

### Quenching and extraction of intracellular metabolites

Quenching and metabolite extraction were carried out as previously described (Winder et al. 2008), with some modifications. In brief, culture broths were injected rapidly into 4 volumes of 60% aqueous methanol solution (−40°C) for quenching. Supernatants after centrifugation at 3000 × *g* at −9°C for 10 min for quenching were removed rapidly, and washed with 1 ml of 60% aqueous methanol (−40°C), followed by centrifugation at 3000 × *g* at −9°C for 10 min. Subsequently, supernatants were thoroughly removed, and cell pellets were frozen in liquid nitrogen and kept at −80°C until the following extraction procedures. Cell pellets were suspended in 500 μl of 100% methanol (−40°C), frozen in liquid nitrogen, and allowed to thaw on dry ice. After, the freeze-thaw cycle was performed 3 times in total, the suspensions were centrifuged at 16000 × *g*, at −9°C, for 5 min. Supernatants were retained and stored on dry ice, and another aliquot (500 μl) of 100% methanol (−40°C) was added to each pellet. The procedure was repeated twice, and the second aliquot of methanol was combined with the first one.

### Metabolite derivatization

Extract aliquots and cultivation medium supernatants (20 μl each), as well as dilution series of standard mixtures of target metabolites (Table 1), were spiked with internal standards (ribitol, 10 or 1 μg for the extracellular or intracellular analysis, respectively) and lyophilized. Dried samples were subsequently derivatized in 2 stages, as previously described (Tsugawa et al. 2011). For oximation, 100 μl (50 μl for intracellular metabolites) of methoxyamine hydrochloride (Sigma-Aldrich, St. Louis, MO) in pyridine (20 mg/l) (Wako, Tokyo, Japan) was added and incubated at 30°C for 90 min. For trimethylsilylation, 50 μl (25 μl for intracellular metabolites) of *N*-methyl-*N*-(trimethylsilyl)trifluoroacetamide (GL Science, Tokyo, Japan) was added and incubated at 37°C for 30 min. Insoluble residues were removed by centrifugation at 12000 × *g* at 4°C for 5 min, and cultivation supernatants were transferred to clean vials.

**Table 1:** Target metabolites detected by GC/MS

**Name**	**Retention time (min)**	**Formula**	***m/z *** **Range**
Pyruvate + OAA	6.34	C_6_H_12_NO_3_Si	174–177
Lactate	6.72	C_8_H_19_O_3_Si_2_	219–222
Succinate	14.45	C_9_H_19_O_4_Si_2_	247–251
Fumarate	15.31	C_9_H_17_O_4_Si_2_	245–249
Malate	17.42	C_12_H_27_O_5_Si_3_	335–339
PEP	18.39	C_11_H_26_O_6_PSi_3_	369–372
Citrate	19.79	C_17_H_37_O_7_Si_4_	465–471
Ribitol (i.s.^a^)	19.32	C_19_H_49_O_5_Si_5_	219

(i.s.^a^), internal standard.

### GC/MS analysis and data processing

Derivatized metabolites were analyzed using GCMS-QP2010 Ultra (Shimadzu, Kyoto, Japan) equipped with a 30 m × 0.25 mm i.d. fused silica capillary column coated with 0.25-μm CP-SIL 8 CB low bleed (Agilent Technologies, Santa Clara, CA). Aliquots (1 μl) were injected in the split mode (25/1, supernatant analysis; 5/1, intracellular analysis) at 230°C, using helium as carrier gas at a flow rate of 1.12 ml/min. The column temperature was held at 80°C for 2 min isothermally, raised to 130°C (4°C/min) and then to 330°C (25°C/min), and maintained for 6 min isothermally. The interface and MS source temperatures were 250°C and 200°C, respectively, and the ion voltage was 1 kV. Data were collected by GCMS solution (Shimadzu), and identified metabolites are shown in Table 1. Mass isotopomer distributions were corrected for natural isotope abundance as previously described (Nanchen et al. 2007). The GC/MS analysis was performed on 3 biological replicates of each sample.

## Results

### CO_2_ incorporation into *C. cellulovorans* metabolites

**Figure 2:**
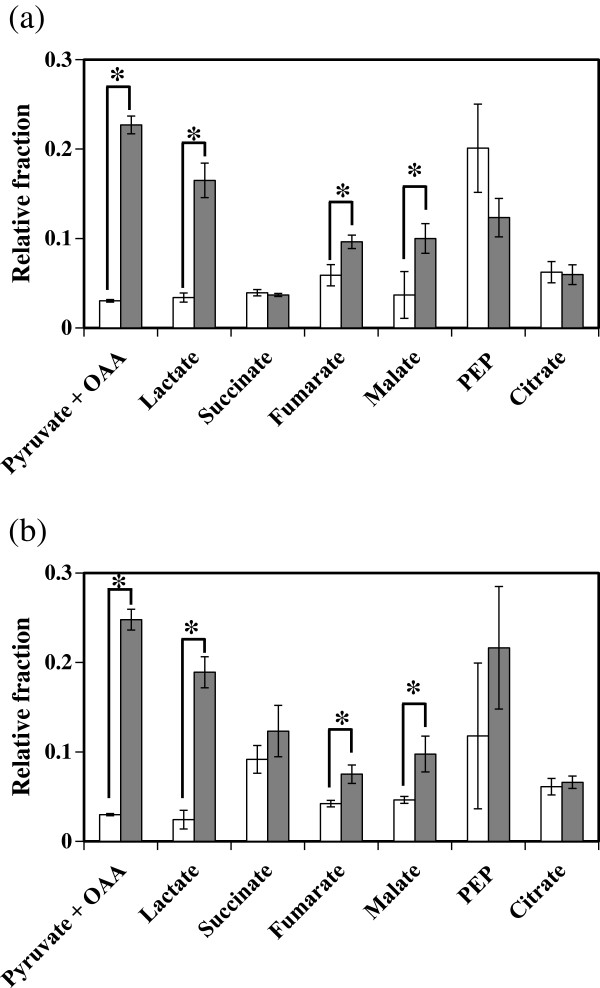
**Incorporation of CO**_**2 **_**into several metabolites inside and outside of the cells.** The vertical axis represents the relative fraction of ^13^C-labeled metabolites, which was calculated as follow as: *m*_1_/ $\sum_{i=0}^{n}m_{i}$, where *m*_*1*_ and *m*_*i*_ are the corrected intensities and *n* is the number of carbon atoms in metabolites (*n* = 3 in the case of lactate, for example). **(a)** Intracellular metabolites. **(b)** Extracellular metabolites. White and gray bars represent the ratios of each metabolite in media containing NaHCO_3_, and NaH^13^CO_3_, respectively. Error bars and asterisks indicate standard deviations and significant difference (*P < 0.01), respectively.

According to previous reports that CO_2_ was required for culturing some *Clostridium* species, we speculate that *C. cellulovorans* also has the activity of CO_2_ fixation. Our speculation is further supported by the fact that *C. cellulovorans*, whose genes related to CO_2_ fixation were also annotated in the genome of *C. cellulovorans* (Tamaru et al. 2010a), can be cultivated in media containing higher CO_2_ concentrations, even at ‘100%’, compared to other *Clostridium* species. Therefore, in this study, we cultivated *C. cellulovorans* in media containing NaH^13^CO_3_ instead of NaHCO_3_ and then examined a massive number of metabolites derived from *C. cellulovorans* and cultivation supernatants using GC/MS. Figure 2 shows the ratios of each metabolite in media containing either NaHCO_3_ or NaH^13^CO_3_. Higher values of relative fractions when *C. cellulovorans* was cultivated in media containing NaH^13^CO_3_ indicate that ^13^C atoms derived from NaH^13^CO_3_ were incorporated into specific metabolites. Our results demonstrate that when *C. cellulovorans* was cultivated in media containing NaH^13^CO_3_, the relative fractions of pyruvate + oxaloacetate (OAA), lactate, fumarate, and malate inside (Figure 2a) and outside (Figure 2b) cells were significantly higher than those from *C. cellulovorans* cultivated in media containing NaHCO_3_. Based on these findings, *C. cellulovorans* evidently is able to incorporate ^13^C atoms into abovementioned metabolites, and therefore has the ability to fix CO_2_.

### Glucose metabolism into metabolic pathway intermediates

**Figure 3:**
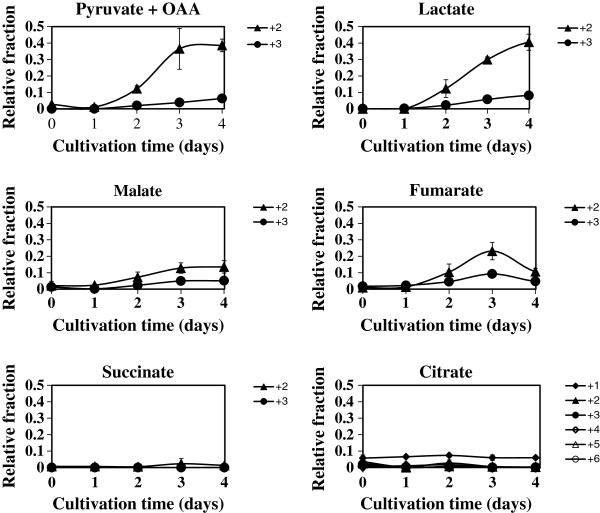
**Dynamic incorporation of [U-**^**13**^**C]-glucose into target metabolites.** The abscissa and vertical axes represent the cultivation time of *C. cellulovorans* and the relative fraction of indicated labeled compounds. ' + 1', ' + 2', ' + 3', ' + 4', ' + 5', and ' + 6' mean the number of 13-carbon isotope in each metabolite incorporated from [U-^13^C]-glucose. Error bars represent standard deviations.

Next, to understand the whole strategy of glucose metabolism in *C. cellulovorans*, we examined a massive number of metabolites inside bacterial cells that were cultivated in media containing [U-^13^C]-glucose. In this way, there is a report how metabolites flow in metabolic pathway of *C. acetobutylicum* have been analyzed (Amador-Noguez et al. 2010). To more understand metabolites flow in *C. cellulovorans*, we observed how ^13^C atoms were incorporated into some metabolites. The results shown in Figure 3 indicate that ^13^C atoms derived from [U-^13^C]-glucose were incorporated into pyruvate + OAA, lactate, fumarate, and malate inside the cells. These results also demonstrate the following 4 points. First, both PFOR and PEPC fixed CO_2_. It is because that pyruvate had only two ^13^C atoms of three carbons (Figure 3). The results indicated that pyruvate was converted from acetyl-CoA associated with CO_2_ fixation once pyruvate became acetyl-CoA, which is constructed two carbons in acetyl group. In the same way, as malate and fumarate had three ^13^C atoms, they could be prepared from PEP by PEPC associated with CO_2_ fixation. Second, PFOR initiated the reversible conversion of pyruvate to acetyl-CoA. Third, the amount of PEP flowing into the TCA cycle could be much less than that flowing into pyruvate, acetyl-CoA, and lactate. Fourth, under this condition, ^13^C atoms were not incorporated into succinate and citrate.

### Lactate secretion accompanied with CO_2_ fixation

**Figure 4:**
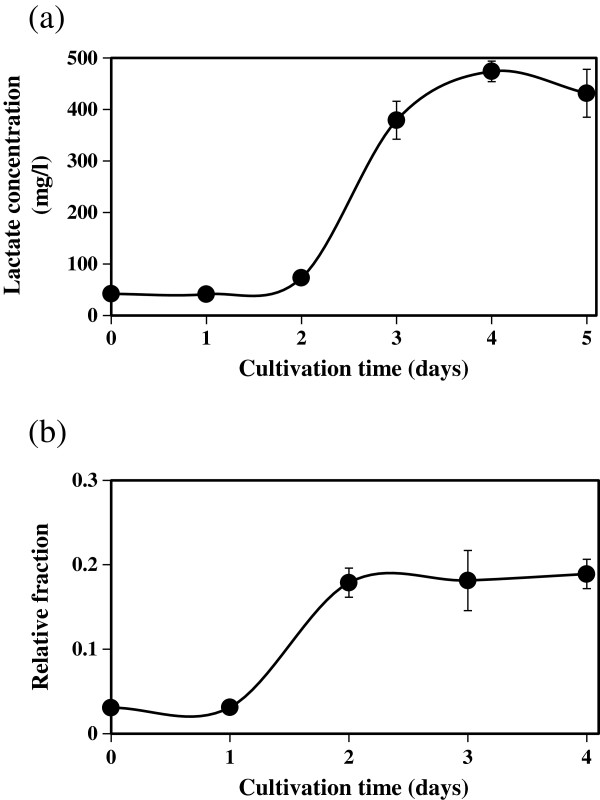
**Lactate secretion accompanied with CO**_**2 **_**fixation. (a)** The time course of lactate concentration in cultivation supernatants. **(b)** The time course of relative fraction of lactate in cultivation supernatants. Relative fractions were calculated as described in Figure 2. Error bars represent standard deviations.

As shown in Figures 2 and 3, a flux of lactate was observed in *C. cellulovorans*, in agreement with the previous report (Sleat et al. 1984). Therefore, we checked the amount of secreted lactate by *C. cellulovorans* cultivated in media containing NaH^13^CO_3_ (Figure 4a). We further calculated the percentage of ^13^C incorporation into secreted lactate. The results show that, accompanied with CO_2_ fixation, *C. cellulovorans* produced lactate at a constant rate after 2 days (Figure 4b).

## Discussion

**Figure 5:**
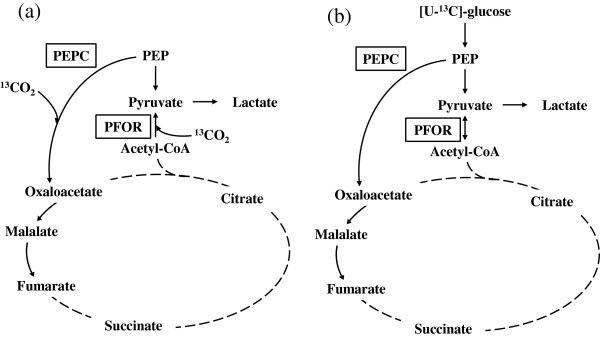
**Proposed pathway for CO**_**2 **_**incorporation in *C. cellulovorans*.** Possible metabolic maps of *C. cellulovorans* in media containing NaH^13^CO_3_**(a)** and [U-^13^C]-glucose **(b)**. Solid lines, possible pathways with directions indicated by arrows; dashed lines, impossible pathways in our studies. Lactate is produced from pyruvate, whereas fumarate is generated from oxaloacetate and malate. CO_2_ is fixed by PFOR and PEPC. In contrast, no citrate is produced from oxaloacetate.

Using target metabolomics, we demonstrate here that *C. cellulovorans* produces lactate, malate, and fumarate. As illustrated in the metabolic map, including the TCA cycle of *C. cellulovorans* (Figure 5), we propose that *C. cellulovorans* produces lactate accompanied with CO_2_ fixation and generates fumarate by partly operating the TCA cycle in a reductive manner (Figure 5a). The reason why *C. cellulovorans* operates these metabolic pathways (lactate and fumarate production and CO_2_ fixation, except for the PEPC reaction) could be the preservation of redox balance in the cell. That is, the reactions of lactate and malate production (operated by lactate dehydrogenase and malate dehydrogenase, respectively) might be accompanied with the regeneration of 1 molecule of NAD(P)^+^. In addition, the reaction of CO_2_ fixation by PFOR produces oxidized ferredoxin, and a molecule of oxidized ferredoxin subsequently produces 2 molecules of NAD^+^. These reactions may help oxidizing agents to be used in glycolysis. We also examined the existence of CO_2_ fixation enzymes (PFOR and PEPC) by the proteome analysis (data not shown). Compared to the flux from glycolysis to the TCA cycle, the flux to lactate could be dominant, since our results show that higher amounts of ^13^C atoms were incorporated into lactate, but not malate and fumarate (Figure 3).

Our findings also indicate that little citrate and succinate was produced from glucose (Figure 3). Isocitrate dehydrogenase, which operates downstream of citrate in the TCA cycle and operates in an oxidative manner with NAD(P)^+^, could not be used. It is known that citrate is produced from glutamate in some organisms. The metabolic information of *C. acetobutylicum* (Amador-Noguez et al. 2010) also suggested that *C. cellulovorans* could use amino acids (glutamate/glutamine) to make other metabolites. If *C. cellulovorans* produces citrate from glutamate, redox balance would be better maintained because glutamate dehydrogenase or glutamate synthase uses NADP^+^ and isocitrate dehydrogenase uses NADPH. In particular, when *C. cellulovorans* lives under a reductive condition, such a pathway is more reasonable than the pathway of citrate production from acetyl-CoA in an oxidative manner. To examine this hypothesis in the future investigation, it will be a promising approach to study how ^13^C atoms are incorporated into metabolites when using media containing ^13^C-labeled glutamate. Some other amino acids may be needed to maintain the metabolic pathway in *C. cellulovorans*, because the bacterium cannot be cultivated in media without yeast extract, which has glutamate (Sleat et al. 1984).

As mentioned above, we speculate that *C. cellulovorans* could use the mechanism to maintain redox balance, because the oxidizability (the ability to oxygenate other metabolites) is valuable for the condition which was absent from O_2_. *C. cellulovorans* lives under anaerobic conditions because photosynthesis is not operated under the natural growth condition of *C. cellulovorans* (wood chip). It has been reported that CO_2_ fixation is useful to maintain redox balance in microorganisms that have the Calvin cycle or TCA cycle (McKinlay and Harwood 2010); therefore CO_2_ fixation could be a common mechanism to regulate redox balance.

Notably, lactate production in cultivation supernatants after 4 days was 474 mg/l (= 31.6 mol per 100 mol glucose), which is about twice of that reported in the previous study (Sleat et al. 1984), where cellobiose was used as a carbon source.

Here, we demonstrated that, accompanied with CO_2_ fixation, *C. cellulovorans* produced several kinds of organic acids and that the TCA cycle was partly operated in a reductive manner at the metabolite level. These presented results would provide important information for the application of *C. cellulovorans* as industrial cellulosome-producing bacteria.

## Competing interests

The authors declare that they have no competing interests.

## Authors’ contributions

MS, HM, HM, YT, EF, KK and MU conceived and designed the study. MS, HS, HM and EF analyzed the data. MS, HS, HM, KK and MU drafted the manuscript. All authors read and approved the final manuscript.
